# Dissertation on the Methods of Degrading the Dental Profession

**Published:** 1842-09

**Authors:** 


*Dissertation on the Methods of Degrading the Dental Profession.*—The editors have concluded that inasmuch as this essay is to be published by private subscription, three thousand three hundred copies having already been ordered, there would be no necessity for publishing it in the Journal, agreeably with the resolution of the society to that effect. It will probably be seen by all our subscribers in pamphlet form. Its place therefore in the Journal may be given to other matter. The editors trust that they shall be justified by the society for this departure from their instructions. Persons wishing copies of the above dissertation will be supplied with any number they may order by application to either of the editors of this Journal.

